# Rapid Identification Method for CH_4_/CO/CH_4_-CO Gas Mixtures Based on Electronic Nose

**DOI:** 10.3390/s23062975

**Published:** 2023-03-09

**Authors:** Jianxin Yin, Yongli Zhao, Zhi Peng, Fushuai Ba, Peng Peng, Xiaolong Liu, Qian Rong, Youmin Guo, Yafei Zhang

**Affiliations:** 1 School of Mechanical and Automotive Engineering, Shanghai University of Engineering Science, Shanghai 201620, China; 2 School of Materials, Sun Yat-sen University, Shenzhen 518107, China; 3 School and Materials Science and Technology, Anhui University, Hefei 230601, China

**Keywords:** electronic nose, gas identification, CH_4_-CO, gas mixtures

## Abstract

The inherent cross-sensitivity of semiconductor gas sensors makes them extremely challenging to accurately detect mixed gases. In order to solve this problem, this paper designed an electronic nose (E-nose) with seven gas sensors and proposed a rapid method for identifying CH_4_, CO, and their mixtures. Most reported methods for E-nose were based on analyzing the entire response process and employing complex algorithms, such as neural network, which result in long time-consuming processes for gas detection and identification. To overcome these shortcomings, this paper firstly proposes a way to shorten the gas detection time by analyzing only the start stage of the E-nose response instead of the entire response process. Subsequently, two polynomial fitting methods for extracting gas features are designed according to the characteristics of the E-nose response curves. Finally, in order to shorten the time consumption of calculation and reduce the complexity of the identification model, linear discriminant analysis (LDA) is introduced to reduce the dimensionality of the extracted feature datasets, and an XGBoost-based gas identification model is trained using the LDA optimized feature datasets. The experimental results show that the proposed method can shorten the gas detection time, obtain sufficient gas features, and achieve nearly 100% identification accuracy for CH_4_, CO, and their mixed gases.

## 1. Introduction

CH_4_ and CO are common dangerous gases in industrial production and daily life, which often cause explosions, fires, poisoning, and other accidents. In order to effectively prevent such accidents, it is necessary to carry out rapid and accurate identification for CH_4_, CO, and their mixed gases. At present, the common detection methods are mass spectrometry, light spectrum, gas chromatography, and a chemical method, which can accurately identify the gas composition and concentration [1,2,3,4]. However, their disadvantages, such as high cost, huge equipment, complicated operations, and time-consuming analysis greatly limit their applications. In contrast, semiconductor gas sensors have been widely used for gas detection due to their advantages of small volume, high sensitivity, low power consumption, and real-time detection [5,6].

However, semiconductor gas sensors have inherent cross-sensitivity to different kinds of gases. It is therefore impossible to accurately detect and identify the composition and concentration of the mixed gas by using only one semiconductor gas sensor [7]. By combining different types of gas sensors into a sensor array, the multi-dimensional information of the target gas could be obtained so that the detection and identification of complex atmosphere could be realized [7,8,9]. Electronic nose (E-nose) is a system designed based on this principle to mimic the biological olfactory function. It has been applied in many fields, such as agriculture and the food industry [10], the environment [11], and disease diagnosis [9]. When CH_4_, CO, or their mixed gases exist in the ambient air, it is expected to realize the detection and identification of such gases by using E-nose.

Although many research studies on the detection and identification of CH_4_ and CO have been carried out using E-nose, there are still some problems: (1) Most previous studies mainly focused on investigating single gases [12,13], but there are often mixed gases in the actual environment, so these studies cannot meet the requirement of practical applications. (2) The reported research on gas identification through E-nose is usually based on analyzing the entire response process of E-nose, which makes the detection time relatively long, meaning it cannot meet the requirements of rapid detection [14,15]. (3) Most studies that achieve high identification accuracies require running complex algorithms (e.g., neural network algorithm) [15,16], which limit the application of this technique to portable products.

In order to solve the abovementioned problems of using E-nose to identify gases, in this paper, an E-nose system with seven homemade semiconductor gas sensors was first designed and then used to detect CH_4_, CO, and their mixed gases with different composition and concentrations. In order to improve the detection speed, this paper proposes a feature extraction method based on the start stage of the E-nose response so that the detection process no longer needs to wait for the sensors to reach stable state. Subsequently, two polynomial fitting methods for extracting gas features are designed according to the characteristics of the E-nose response curves. Finally, in order to shorten the time consumption of the calculation and reduce the complexity of the identification model, linear discriminant analysis (LDA) is introduced to reduce the dimensionality of the extracted feature datasets, and the XGBoost-based gas identification model is trained using the LDA optimized feature datasets. The experimental results show that the proposed method can obtain sufficient features representing the target gas, shorten the gas identification time, and achieve high accuracy for CH_4_, CO, and their mixed gases.

## 2. Methodology of Gas Identification

**Figure 1:**
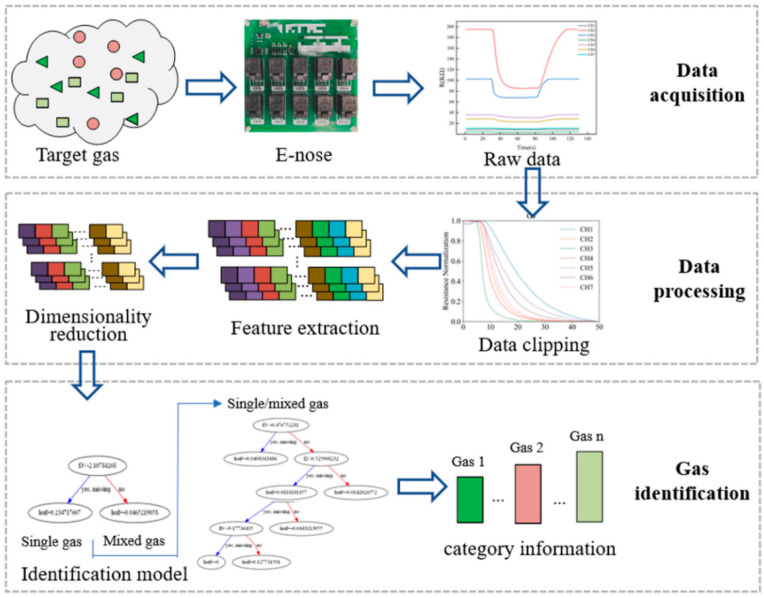
Flowchart of the proposed gas identification method.

Figure 1 illustrates the flowchart of the gas identification method proposed in this paper, which consists of three main processes: data acquisition, data processing, and gas identification. The data acquisition process obtains the response of the E-nose to the target gas. However, the original response may contain noise information, which may come from electromagnetic interference, signal transmission failure, and the fluctuation of the experimental environment. The purpose of data preprocessing is to filter out the noise and interference information from the collected response signal. For training the identification model, as many as possible key features that can represent the target gas need to be first extracted. The feature dataset composed of these preliminary extracted features is normally of high dimensionality. In order to shorten the time consumption of the calculation and reduce the complexity of the recognition model, the feature dataset needs to be optimized by dimensionality reduction algorithms. Finally, in order to realize the identification of the target gas, the optimized feature dataset is used to train the gas identification model.

In order to reduce the complexity of the identification model, an identification model based on the XGBoost algorithm is proposed in this paper. XGBoost is an improvement based on the gradient boosting algorithm proposed by Friedman [17], which has been proven to be highly effective in the fields of medicine [18] and material and fault detection [19,20]. However, there are still few reported applications on gas identification. The specific execution logic of the gas identification algorithm based on XGBoost is as follows:

Assume that a given feature dataset consists of *m* gas samples, and each sample has *n* features. The XGBoost model is considered an integrated model composed of *t* base models, and one base model represents a tree (gases with different categories), so the predicted value of the XGBoost model to identify the *m* gas samples can be expressed as:

(1) $${\hat{y}}_{i}^{\left(t\right)}=\overset{t}{\underset{k=1}{\sum }}f_{k}\left(x_{i}\right)$$

where *x_i_* is the features of gas sample, and *f_k_* is the predicted value of the *k*th tree model. We iterate each tree to obtain the optimal $\hat{y}$ by fitting the residual difference of the current model. The objective function is given by Equation (2), and the goal of XGBoost model is to minimize the value of *obj*.

(2) $$obj=\overset{m}{\underset{i=1}{\sum }}l\left(y_{i},{\hat{y}}_{i}\right)+\overset{t}{\underset{k=1}{\sum }}\Omega \left(f_{k}\right)$$

where $l\left(y_{i},{\hat{y}}_{i}\right)$ is the loss function to measure the deviation between the real value $y_{i}$ and the predicted value ${\hat{y}}_{i}$, and $\sum_{k=1}^{t}\Omega \left(f_{k}\right)$ is a regularization term to balance the complexity of the model. Ω(f) could be calculated as the following equation:

(3) $$\Omega \left(f\right)=\gamma T+\frac{1}{2}\lambda \overset{T}{\underset{j=1}{\sum }}\omega_{j}^{2}$$

where *ω_j_* is the value of the leaf node (gas samples with the same category), *T* is the number of leaf nodes, and *λ* and *γ* represent the penalty coefficient. In the process of generating the *f*th tree, the predicted value and the objective function can be represented by Equations (4) and (5), respectively:

(4) $${\hat{y}}_{i}^{\left(f\right)}={\hat{y}}_{i}^{\left(f-1\right)}+f_{f}\left(x_{i}\right)$$

(5) $$obj^{\left(f\right)}=\overset{m}{\underset{i=1}{\sum }}l\left(y_{i},{\hat{y}}_{i}\right)+\overset{f}{\underset{k=1}{\sum }}\Omega \left(f_{k}\right)$$

Substitute Equation (4) into Equation (5), and the objective function is then approximated by a second-order Taylor expansion, and we find the *f_f_* that minimizes the objective function, as shown in Equation (6):

(6) $$obj^{\left(f\right)}\approx \overset{m}{\underset{i=1}{\sum }}\left[l\left(y_{i},{\hat{y}}^{\left(f-1\right)}\right)+g_{i}f_{f}\left(x_{i}\right)+\frac{1}{2}h_{i}f_{f}^{2}\left(x_{i}\right)\right]+\Omega \left(f_{f}\right)$$

where *g_i_* and *h_i_* are the first and second derivatives of the loss function, respectively. Therefore, the optimal value of leaf *j* ($w_{j}^{*}$) can be calculated by Equation (7), and the predicted value *f_f_*(*x_i_*) of the *f*th tree can be expressed as $w_{q\left(x_{i}\right)}$.

(7) $$w_{j}^{*}=-\frac{\sum_{i\in l_{j}}g_{i}}{\sum_{i\in l_{j}}h_{i}+\lambda }$$

where *q*(*x_i_*) indicates which leaf node the sample *x_i_* falls on, and $i\in l_{j}$ represents the sample number of leaf node *j*. The optimal value of the objective function can be calculated by Equation (8):

(8) $$obj^{\left(f\right)}\approx -\frac{1}{2}\overset{T}{\underset{j=1}{\sum }}\frac{{\left(\sum_{i\in l_{j}}g_{i}\right)}^{2}}{\sum_{i\in l_{j}}h_{i}+\lambda }+\gamma T$$

Calculate the information gain of each gas feature according to Equation (9), and select the branch with the largest gain to construct the model.

(9) $$\mathrm{gain}=\frac{1}{2}\left[\frac{{\left(\sum_{i\in l_{L}}g_{i}\right)}^{2}}{\sum_{i\in I_{L}}h_{i}+\lambda }+\frac{{\left(\sum_{i\in I_{R}}g_{i}\right)}^{2}}{\sum_{i\in I_{R}}h_{i}+\lambda }-\frac{{\left(\sum_{i\in I}g_{i}\right)}^{2}}{\sum_{i\in l}h_{i}+\lambda }\right]-\gamma$$

where $i=l_{L}\cup l_{R}$, *l_L_*, and *l_R_* are the sample sets of the left and right branch after splitting the leaf nodes.

According to the above principles, *t* trees can be constructed, and all the trees are combined to form the identification model. For the sample classified into a leaf node, its final prediction result can be obtained by accumulating the value $w_{j}^{*}$ of all the corresponding leaf nodes.

## 3. Materials and Methods

### 3.1. Design of E-Nose System

**Figure 2:**
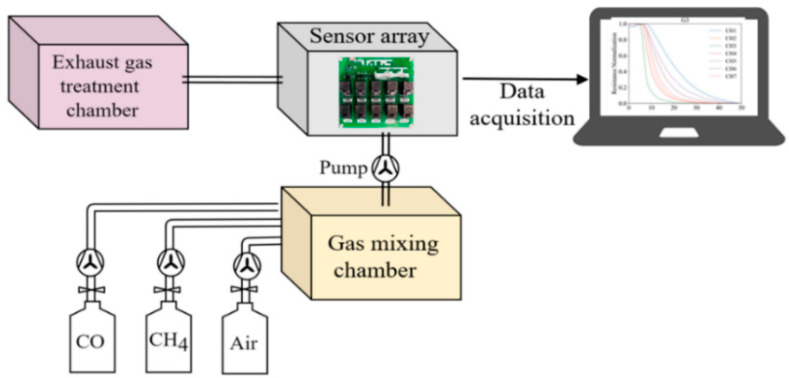
Schematic diagram of the E-nose system.

The E-nose system designed in this paper consists of a gas delivery module, a sensor array, a signal acquisition module, and pattern recognition algorithms, as shown in Figure 2. The gas delivery module delivers different gases into the gas mixing chamber through pumps and then delivers the mixed gas to the chamber where the sensor array is located (volume: 2 L). Each channel of the sensor array is independent, and each sensor transmits its response data separately to the computer.

**Figure 3:**
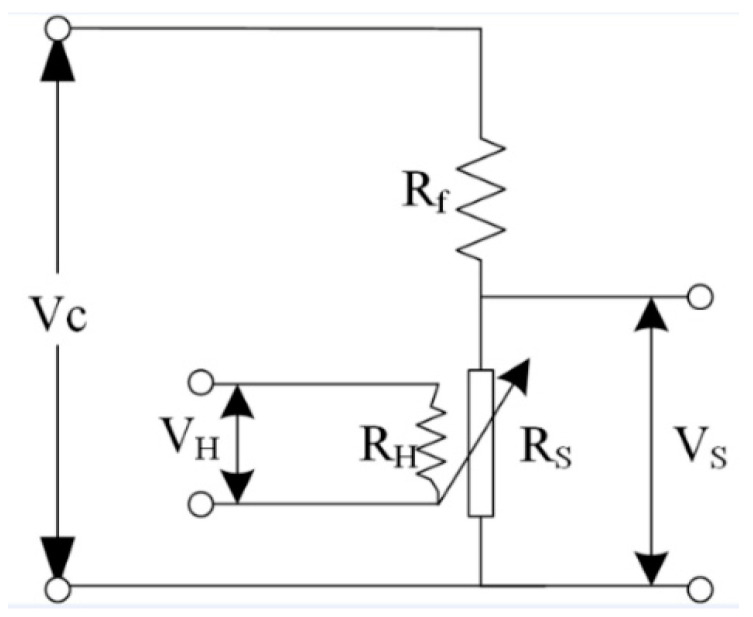
Schematic of detection circuit of the gas sensor.

In this study, the selection of sensors mainly considers two indicators: (1) each sensor can respond to all target gases; (2) each sensor has different response sensitivities to different gases. Seven self-made metal oxide semiconductor gas sensors were selected to form the sensor array of the E-nose. These sensors have a multilayer structure and were fabricated by a thin film technique. Firstly, a composite layer of silicon nitride and silicon oxide with about 3 μm was deposited on the silicon wafer as a supporting layer. Subsequently, a platinum microheater was placed on the supporting layer (power consumption is about 30 mW under the heating voltage 1.8 V, corresponding to the working temperature of 350 °C). Finally, metal oxide-based sensitive materials (SnO_2_, etc.) were deposited on an interdigitated Au electrode, which is used to collect gas response signals. To avoid electrical contact between the heater and the interdigital electrode, a layer of insulating material was therefore added between them. The specific characteristics of the seven sensors are listed in Table 1. Figure 3 shows the detection circuit of the gas sensor. The voltage divider circuit is connected in series with the gas sensor *R_s_* and a fixed value resistor *R_f_*. When the sensor senses the target gas, its resistance will be changed, and the changed value is used as the response signal of the E-nose system.

### 3.2. Experimental Procedure

The as-designed E-nose system was used to detect and identify various types of atmospheres containing CH_4_, CO, and their mixed gases. The composition and concentration of the experimental sample gases are listed in Table 2. Each type of gas was tested 10 times, and a total of 150 samples were collected. The temperature of the experimental environment was 25 ± 1 °C with humidity 60 ± 5%. The detailed steps of the experiment are as follows:

- Sensor preheating: preheat the sensor array before the detection, clean the sensor array with ambient air, and wait for the sensor to reach the stable baseline. This process takes about 30 s.
- Gas sampling: the gas to be detected is introduced to the sensor array chamber for 10 s, and the response signal of the sensor array is collected in real time.
- Sensor recovery: purge sensor array chamber with ambient air more than 90 s. After all sensors return to the initial baseline state, the next gas detection will be performed.

## 4. Results and Discussion

### 4.1. Response of the E-Nose

Affected by the characteristics of the sensitive material of the sensor, the steady-state value of different sensors will change with some factors, such as ambient atmosphere, temperature, and humidity. It may be that the absolute change value of the sensor resistance is small, but its relative change rate is very large, or vice versa. In order to avoid the impact of such a situation on the subsequent gas detection and identification, we use Equation (10) to convert the original data collected by E-nose.

(10) $$R_{s}=\frac{R_{0}-R_{t}}{R_{0}}\;$$

where *R*_0_ is the steady-state resistance of the sensor in the air, and *R_t_* is the real-time resistance of the sensor when it is exposed in the target gas.

**Figure 4:**
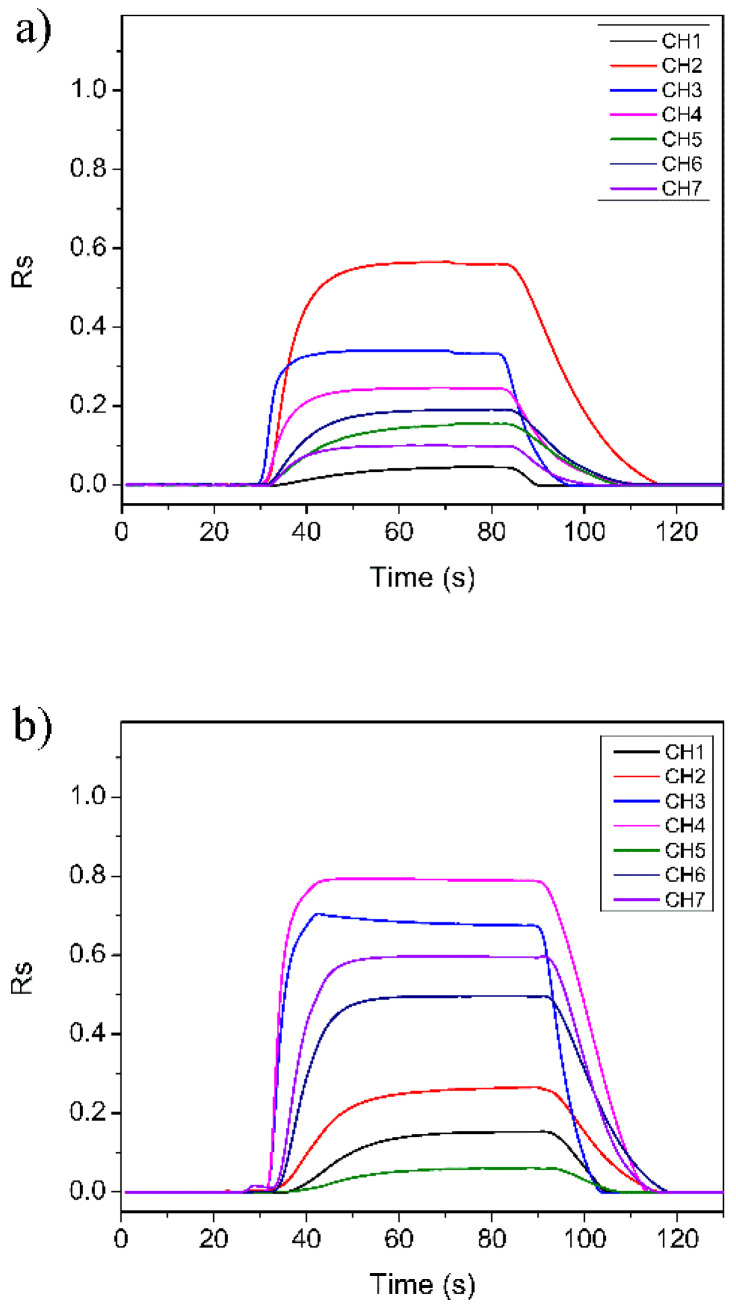

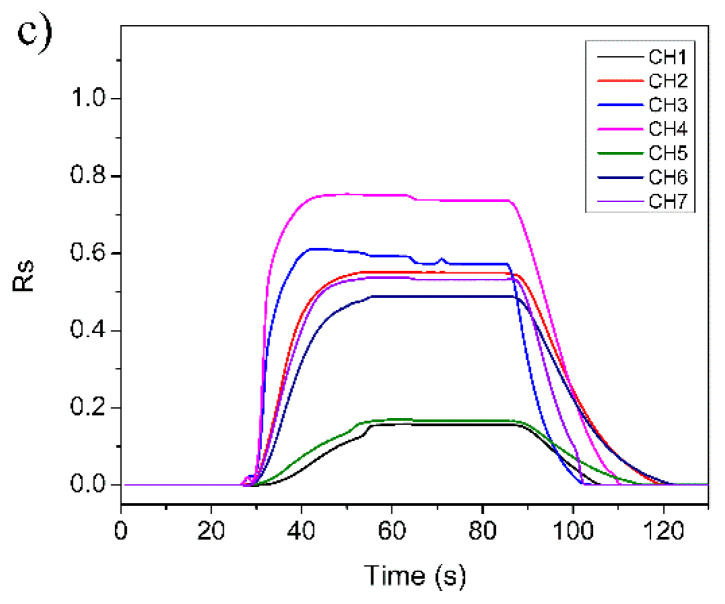
Typical response curve of the E-nose to the detected gases: (**a**) gas G3; (**b**) gas G6; (**c**) gas G15.

Figure 4 shows the typical response spectrum of the E-nose to the detected gases converted by Equation (10). As the sensors of the E-nose system are n-type SMO and tested gases are reducing gases, the *R_s_* values of all sensors are positive. It can be seen that when the target gas was sent into the sensor array chamber, all the sensors exhibited fast responses. Their responses reached the maximum value as the target gas was completely injected into the test chamber. For instance, the absolute resistance value of Sensor CH1 in air is about 8.7 kΩ, and its maximum responses in G3, G6, and G15 reduce to 8.3, 8.0, and 7.5 kΩ, respectively. When the clean air was introduced, all the sensors can quickly return to their initial states. This result indicates that the as-designed E-nose system has good detection ability.

An interesting phenomenon can also be found from Figure 4. In the start stage when the target gas was sent into the test chamber, the sensor array showed obvious differences for different types of gases, that is, the start stage of the response curves of different types of gases has good discrimination. The start stage is actually the reaction process between the detection gas and the sensitive material of the sensor [21]. For the experimental gases, this process takes about 15 s (from 30th s to about 45th s). Therefore, we can use the response data of this process to distinguish the gases. In the next section, a feature extraction method based on the start stage of the E-nose response is explained in detail for achieving rapid detection and identification of the gases.

### 4.2. Gas Feature Extraction

At present, most of the feature extraction methods used in the E-nose system are manual, such as calculating the maximum value, minimum value, slope, integral, difference, and derivative of the response curve. However, this method relies on the empirical knowledge of the researcher and requires a large number of tentative experiments. To overcome this shortcoming, this paper proposes a feature extraction method through fitting the start stage of the gas response curve with polynomials. In order to compare the effectiveness of the proposed feature extraction method, we also constructed the manual feature dataset commonly used for E-nose based on previous studies [10,22]. The details of the construction process of each feature dataset are as follows:

#### 4.2.1. Manual Feature Dataset

**Figure 5:**
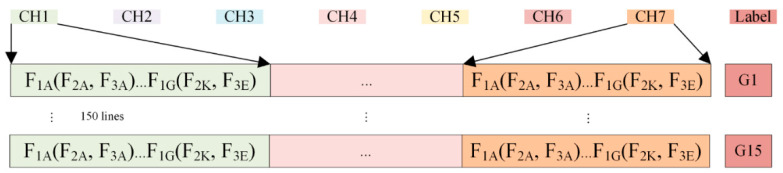
Schematic of the feature vector organization.

As shown in Table 3, the manual feature dataset extracted 24 features from each sensor, and a total of 168 features were obtained for 7 sensors (24 × 7 = 168). The extracted features were sequentially connected to form a feature vector (see Figure 5). Each type of target gas was detected 10 times, and the final feature dataset contained 25,200 features (15 × 10 × 168).

#### 4.2.2. Overall Polynomial Fitting Feature Dataset

**Figure 6:**
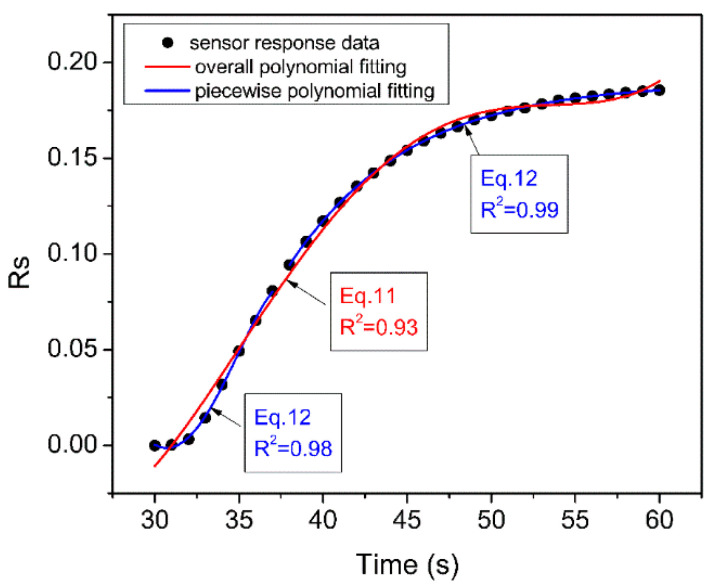
Comparison of feature extraction by overall polynomial fitting and piecewise polynomial fitting.

According to the analysis of the experimental data, the start stage response curves of the CH_4_, CO, and CH_4_-CO gases can be well-fitted by Equation (11), as shown in Figure 6. The polynomial coefficients are considered as the extracted features. Some 11 features were then extracted from each sensor for a total of 77 features for the 7 sensors (11 × 7 = 77). The feature dataset was also organized in the same way as shown in Figure 5, which contained a total of 11,550 features (15 × 10 × 77).

(11) $$y=Ax^{10}+Bx^{9}+\cdots +Jx+K$$

where *y* represents the response value of the gas sensor, *x* is the detection time, and *A, B...* are the fitting coefficients of the polynomial.

#### 4.2.3. Piecewise Polynomial Fitting Feature Dataset

Although Equation (11) can fit the response curve of the sensors well, there are some deviations, especially at both ends of the curve (see Figure 6). Therefore, in order to better match the characteristics of the response curve, we proposed an idea of using piecewise fitting. As illustrated in Figure 6, the response curve was cut into two parts and fitted with two low-order polynomials, respectively, and finally the fitting parameters of the two polynomials were combined to construct the feature dataset. This can not only effectively reduce the calculation of the fitting process, but also hopefully improve the accuracy of the curve fitting. Experimental data showed that each part of the curve could obtain satisfactory fitting accuracy by using a quartic polynomial Equation (12). The coefficients *A*, *B*, *C*, *D*, and *E* are the extracted features. A total of 10 features were extracted from the 2 quartic polynomials, and finally a dataset containing 10,500 features (15 × 10 × 70) was constructed.

(12) $$y=Ax^{4}+Bx^{3}+Cx^{2}+Dx+E.$$

### 4.3. Analysis of Gas Features

The gas feature datasets obtained by the abovementioned three feature extraction processes have very high dimensionalities (higher than 70). In order to shorten the time consumption of the calculation and reduce the complexity of the recognition model, we employed linear discriminant analysis (LDA) to reduce their dimensionalities before the training of the identification model. LDA is a powerful supervised algorithm that computes the linear discriminant by considering the scatter of samples both within classes and between classes.

**Figure 7:**
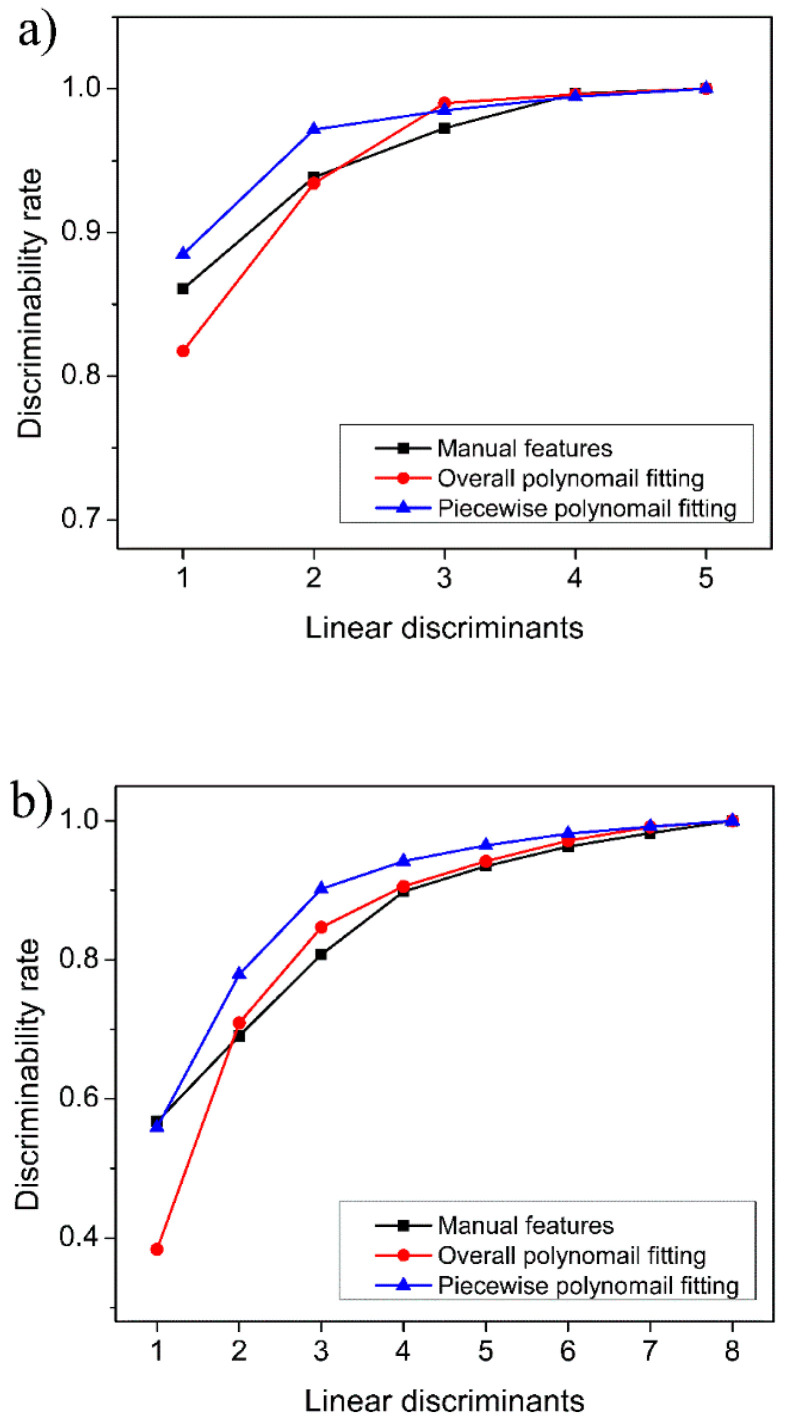
The discriminability rate as a function of linear discriminant numbers: (**a**) single gas; (**b**) mixed gas.

Figure 7 shows the results of LDA dimensionality reduction on the feature datasets established above. It can be seen that, whether for single gases or mixed gases, as the number of discriminants increases, the discriminability rate of samples is significantly improved. For the single gases, higher precision identification of samples can be achieved by retaining three-dimensional features. while for the mixed gases, only about five-dimensional features are required. This result indicates that the feature extraction methods proposed in the previous section are sufficient to represent the gas category information. It also reveals that the identification of mixed gases is more difficult than that of single gases.

**Figure 8:**
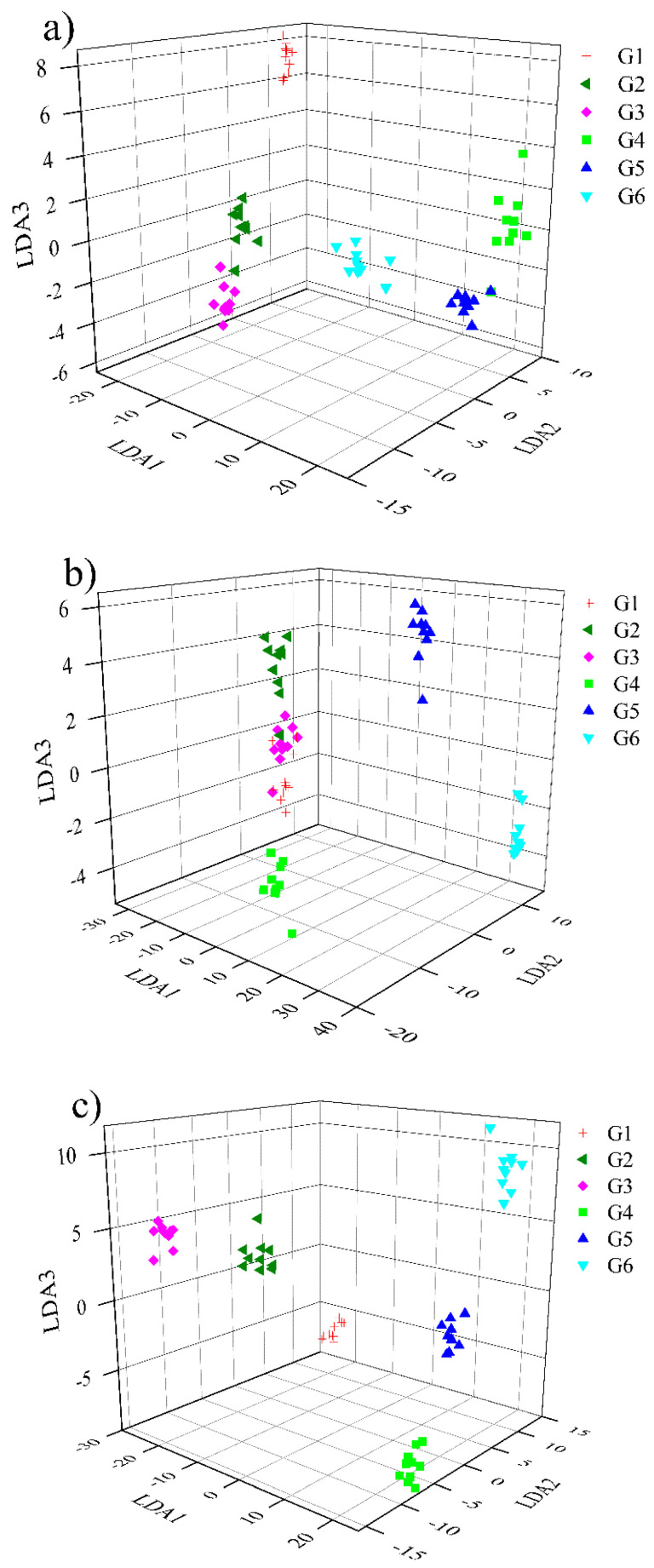
Projection of the first three features reduced by LDA for single gas: (**a**) manual feature; (**b**) overall polynomial fitting feature; (**c**) piecewise polynomial fitting feature.

**Figure 9:**
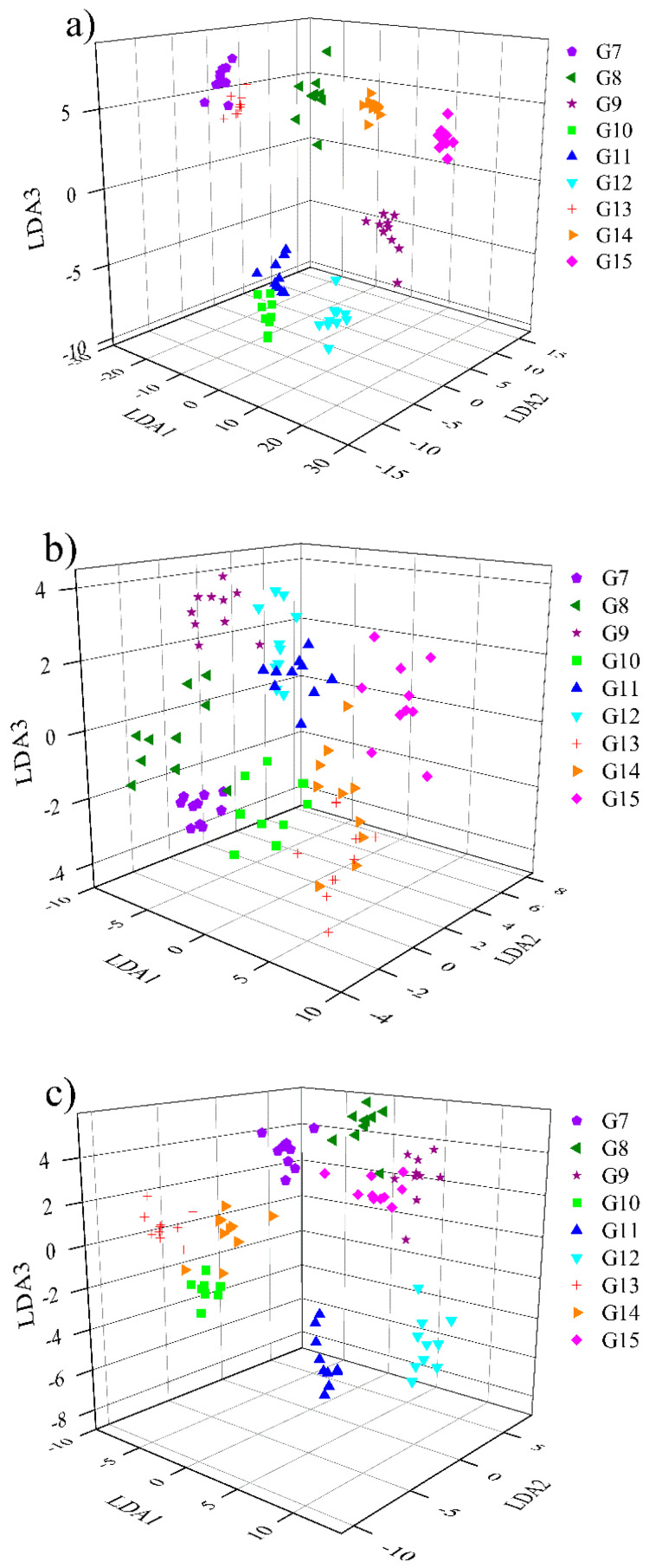
Projection of the first three features reduced by LDA for mixed gas: (**a**) manual feature; (**b**) overall polynomial fitting feature; (**c**) piecewise polynomial fitting feature.

Figure 8 shows the scatter plot of single gas samples after LDA feature dimensionality reduction. It can be seen that the first three features of all feature datasets can clearly distinguish different gas samples. It is worth noting that for overall polynomial fitting features, some samples overlap slightly (gas G1 and G3), which may be caused by the relatively large deviations in the data fitting process (see Figure 6). However, for the mixed gases, it seems that the samples cannot be completely distinguished only through the first three features (see Figure 9). This is because the gas sensor array has cross-sensitivity to both CH_4_ and CO. The responses of the sensors to the mixed gases are therefore more complex than that of the single gases, so those features extracted would be partially overlapped and confused. Although the situation of mixed gases is more complicated, there is an obvious phenomenon: the piecewise polynomial fitting feature dataset is significantly better than the overall polynomial fitting dataset because the samples of the former are better clustered. In contrast, the sample distribution of the latter are more dispersed.

### 4.4. Identification Accuracy

Since the number of features required for the identification of single gases and mixed gases is different, to achieve rapid identification of gas categories, a binary classifier based on XGBoost was first constructed to evaluate whether the sample is a single gas or a mixed gas. Then, further identification will be carried out according to the evaluation results. In order to ensure the accuracy and stability of the XGBoost model, it was trained and verified using a five-fold cross-validation method, in which the subsets for training and verification were randomly generated multiple times.

**Figure 10:**
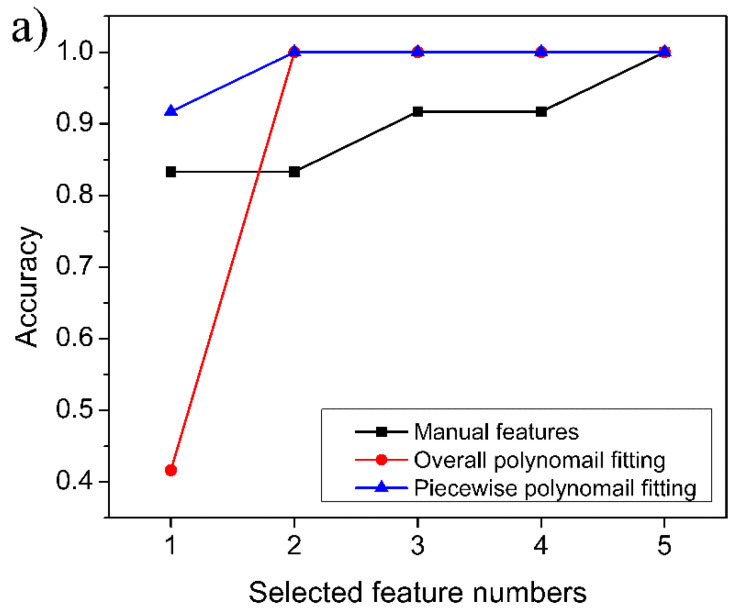

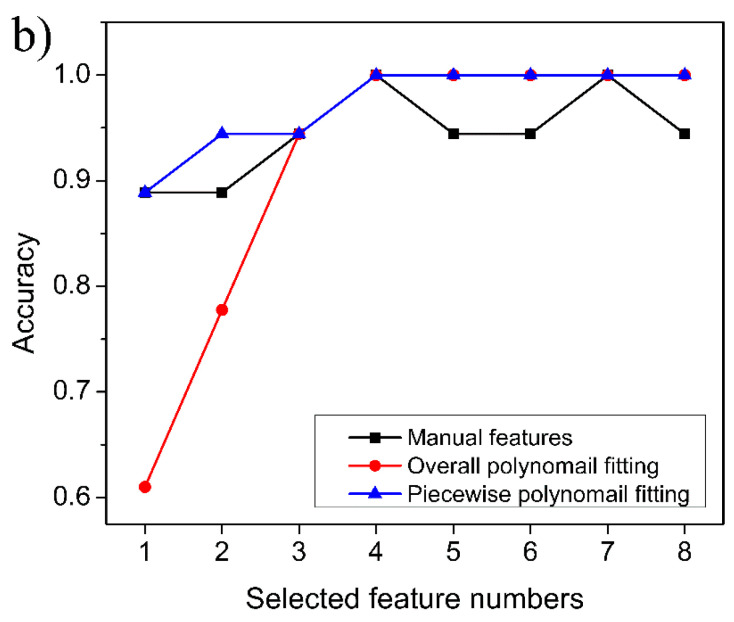
Accuracy of the identification model obtained on verification samples: (**a**) single gas; (**b**) mixed gas.

The experimental results confirmed that the XGboost model is able to determine with complete accuracy whether the target gas is a single gas or a mixed gas. The further experimental results are shown in Figure 10. For the six single gases, the identification accuracy of the manual feature dataset can exceed 80% when two features are retained, and 100% identification accuracy can be achieved with five features. For the overall polynomial fitting feature dataset and the piecewise polynomial fitting feature dataset, 100% accuracy can be obtained when two-dimensional features were used. In particular, for the piecewise polynomial fitting feature dataset, the accuracy of 90% can be achieved with only one-dimensional features. This confirms that the efficiency of feature extraction by piecewise polynomial fitting is highest among the three extraction methods. For mixed gases, the improvement trends of the identification accuracy as a function of the number of features are similar to those of single gas, but the satisfactory identification accuracy requires a larger number of features, that is, four-dimensional features are required (see Figure 10b). Some researchers also reported the detection and identification of CO and CH_4_ gases in recent years [12,15]. The identification accuracies they obtained were around 98%, but these studies employed complex models, such as neural networks. Compared with these previous studies, our proposed method also achieves satisfactory accuracy and is more convenient in terms of identification time consumption.

The experimental results also indicate that the polynomial fitting feature dataset proposed in this paper is more efficient than the manual feature dataset, whether for single gases or for mixed gases. Although the more effective manual feature dataset can be constructed if more tentative experiments can be carried out, this is undoubtedly a laborious work. Therefore, it is more convenient to extract gas features through polynomial fitting. It is worth noting that although both the overall polynomial fitting and the piecewise polynomial fitting could obtain perfect identification efficiency when retaining a sufficient number of features, the piecewise polynomial fitting requires fewer parameters (10 parameters) than that of the overall polynomial fitting (11 parameters). Moreover, the overall polynomial fitting is more time-consuming because of the higher order of Equation (11), so we can draw a conclusion that the piecewise polynomial fitting is more advantageous than the overall polynomial fitting. Therefore, constructing the gas feature dataset by piecewise polynomial fitting, then reducing the dimensionality of the original feature dataset by LDA, and finally constructing the identification model based on the XGBoost algorithm is a rapid and efficient method for identifying CH_4_, CO, and their mixed gases.

## 5. Conclusions

In this paper, the detections of CH_4_, CO, and their mixed gases were carried out by using a homemade E-nose system. Through the analysis of the response signal of the E-nose, a rapid identification method was proposed, which successfully realized the high identification accuracy of the gases with different components and concentrations of CH_4_ and CO. The main results are summarized as follows:

(i) By analyzing the start stage signal of the E-nose response instead of the entire response process, the gas detection time could be effectively reduced.
(ii) Polynomial fitting is an effective gas feature extraction method, and piecewise fitting is more effective than overall fitting.
(iii) The identification model based on the XGboost algorithm can efficiently identify CH_4_, CO, and their mixed gases with high accuracy.

## Figures and Tables

**Table 1:** Characteristics of the employed sensors in E-nose.

Sensor No.	Materials	Main Detected Gas
CH1	ZnO/SnO_2_	VOCs
CH2	Pt/SnO_2_	Carbon monoxide, Air quality control
CH3	Pt/SnO_2_	Hydrogen, Carbon monoxide, Methane
CH4	Pd/SnO_2_	Methane, Hydrogen sulfide, Ethanol
CH5	NiO/SnO_2_	Ethanol, Ammonia
CH6	SnO_2_/MWCNT	Acetone, Hydrogen sulfide, Ethanol
CH7	SnO_2_/MWCNT	Hydrogen sulfide, Acetone, Ethanol

**Table 2:** The composition and concentration of experimental sample gases.

Category	Sample Label	CH_4_ (ppm)	CO (ppm)
Single gas	G1	0	10
	G2	0	20
	G3	0	30
	G4	1000	0
	G5	2000	0
	G6	3000	0
Mixed gas	G7	1000	10
	G8	1000	20
	G9	1000	30
	G10	2000	10
	G11	2000	20
	G12	2000	30
	G13	3000	10
	G14	3000	20
	G15	3000	30

**Table 3:** Extracted gas features by manual method.

Feature Meaning	Function	Feature Number
Maximum difference	$F_{1A}=R_{\max}-R_{\min}$	1
Reaction time	$F_{1B}=t_{\max}-t_{\min}$	1
Integral	$F_{1C}=\int_{t_{\min}}^{t_{\max}}f\left(R_{t}\right)$	1
Response	$F_{1D}=R_{1}\cdots R_{10}$	10
Difference	$F_{1E}=R_{t}-R_{t+1}$	9
Slope	$F_{1F}=\frac{R_{5}-R_{1}}{5}$ $,\;F_{1G}=\frac{R_{10}-R_{6}}{5}$	2

Note: *R* is the resistance of gas sensor, and *t* represents detection time.

## Data Availability

Data is contained within the article.
